# Knowledge and practice on *Toxoplasma* infection in pregnant women from Malaysia, Philippines, and Thailand

**DOI:** 10.3389/fmicb.2014.00291

**Published:** 2014-06-11

**Authors:** Hemah Andiappan, Veeranoot Nissapatorn, Nongyao Sawangjaroen, Si-Lay Khaing, Cristina C. Salibay, Mary Mae M. Cheung, Julieta Z. Dungca, Waenurama Chemoh, Ching Xiao Teng, Yee-Ling Lau, Noor A. Mat Adenan

**Affiliations:** ^1^Department of Parasitology, Faculty of Medicine, University of MalayaKuala Lumpur, Malaysia; ^2^Department of Microbiology, Faculty of Science, Prince of Songkla UniversityHat Yai, Songkhla, Thailand; ^3^Department of Obstetrics and Gynecology, Faculty of Medicine, University of MalayaKuala Lumpur, Malaysia; ^4^Biological Sciences Department, De La Salle University – DasmariñasCavite, Philippines; ^5^College of Arts and Sciences, Notre Dame of Dadiangas UniversityGeneral Santos, Philippines; ^6^School of Science and Technology, Centro Escolar UniversityManila, Philippines

**Keywords:** toxoplasmosis, knowledge, practice, pregnant women, Malaysia, Philippines, Thailand

## Abstract

*Toxoplasma gondii*, is one of the infectious agents of congenital TORCH infections, causes severe clinical outcomes in fetus and newborns. Nevertheless this life-threatening parasitic disease is preventable by simple preventive measures related to lifestyle during pregnancy. We aim to study on the knowledge about toxoplasmosis and practices that prevents this infection among the pregnant women. Total of 2598 pregnant women from Malaysia, Philippines, and Thailand were randomly surveyed to determine the knowledge and their practices on *Toxoplasma* infection. The questionnaire covered respondents' general information and knowledge on plausible risks factors, symptoms, timing of infection, prevention knowledge, and preventive behavior regarding *Toxoplasma* infection. Majority of these pregnant women were in their age group of 20–29 years (50.9%), completed secondary level of education (51.7%), in their second trimester of pregnancies (38.1%), non-parous (36.6%), and had no history of abortion (90.4%). Based on this survey, only 11% of these pregnant women had read, heard, or seen information regarding toxoplasmosis and 3.5% of them were aware of being tested for the infection. A small percentage of these pregnant women knew that *T. gondii* were shed in the feces of infected cats (19.4%) and sometimes found in the raw or undercooked meat (11.0%). There was 16.1% of responding women knew that toxoplasmosis is caused by an infection. Demographic profiles such as age group, level of education, pregnancy term, and number of children of the pregnant women showed significant association with their responses toward prevention knowledge and preventive behavior related questions (*P* < 0.05). Thus, it is suggested that health education on toxoplasmosis and primary behavioral practices should be consistently offered to reproductive age women in general and pregnant women in particular. This information could help to reduce vertical transmission of *Toxoplasma* infection during pregnancy.

## Introduction

*Toxoplasma gondii*, an obligate intracellular coccidian protozoan, is the causative agent of Toxoplasmosis. *Toxoplasma* have three main forms in its entire life cycle, being; oocysts, tachyzoites, and bradyzoites. The definitive host of *Toxoplasma* is the felines, where oocysts are produced in the intestines and passed in feces. The oocyst is infective to humans and other intermediate host (mammals and other warm blooded animals). Once infected, *Toxoplasma* develops itself into tachyzoites (rapid multiplication of *Toxoplasma* in trophozoite form) or maintain dormant in bradyzoites (tissue cysts), depending on the host immune status (John and Petri, 2006). The infection in pregnant women may cause devastating effects in the fetus. If the infected tissue of an animal being consumed, it acts as a transmission mode of infection. Furthermore, this parasitic infection can be transmitted by ingestion of *Toxoplasma* oocysts contaminated fruits and vegetables or unclean water, through blood transfusion, and by receiving organ transplant (Pereira et al., 2010). Exposures to contaminated feces by cleaning cat's litter, gardening, or handling contaminated soils are some of the ways of this parasitic infection being transmitted.

Most countries in South America, Middle Eastern and other low-income countries reported high seropositive for *Toxoplasma* infection from both normal or immunocompromised host, e.g., 59% in Brazil (Ferezin et al., 2013), 84.7% in Congo (Doudou et al., 2014), 3.98% in China (Hua et al., 2013), 83.6% in Ethiopia (Zemene et al., 2012), 75% in India (Chintapalli and Padmaja, 2013), 10.3% in Japan (Sakikawa et al., 2012), 11.8% in Taiwan (Chou et al., 2011) and 30.9% in Tanzania (Mwambe et al., 2013). Meanwhile, the recent seroprevalence rate of *Toxoplasma* infection in pregnant women was reported as 49% in Malaysia (Nissapatorn et al., 2003), 23.8% in the Philippines (Salibay et al., 2008) and 28.3% in Thailand (Nissapatorn et al., 2011).

*Toxoplasma* infection in immunocompetent persons is usually asymptomatic (Halonen and Weiss, 2013) however; infection in the immunodeficient patients and in fetus through their pregnant mother may lead up to severe and often fatal toxoplasmosis. The clinical signs in infected fetus will be intracranial calcifications, hydrocephalus, eye infection, seizures, miscarriage, or death (Paquet and Yudin, 2013). The annual incidence of congenital toxoplasmosis was estimated at 190,100 cases globally (Torgerson and Mastroiacovo, 2013). The clinical presentation was not or rarely shown in infected pregnant women, even during acute infection. Some of the pregnant women may present symptoms like malaise, low-grade fever and lymphadenopathy (Montoya and Remington, 2008). Anti-parasitic antibiotic therapy is the currently available treatment for *Toxoplasma* infected pregnant women (Paquet and Yudin, 2013). The pregnant women were given spiramycin antibiotic if the infection occurs during the first 18 weeks of gestation, meanwhile pyrimethamine, sulfadiazine and folic acid were given to pregnant women who acquired this parasitic infection after 18 weeks of gestation and onwards (Montoya and Remington, 2008). In the meantime, developments of vaccines are still being studied (Verma and Khanna, 2013).

As toxoplasmosis being one of the TORCH [acronym for a group of five infectious disease namely; Toxoplasmosis, Others (Hepatitis B), Rubella (German measles), Cytomegalovirus (CMV), Herpes Simplex Virus (HSV)] infectious agents in pregnant women, knowledge and practice regarding this disease, and preventive measures in lifestyle for this parasitic infection should be given to the pregnant women. To the best of our knowledge, questionnaire base study on toxoplasmosis has never been conducted in pregnant women from this part of the world. Therefore, this survey aimed to evaluate the level of knowledge and practices on toxoplasmosis among pregnant women who visiting the antenatal clinics (ANC) or hospitals from their respective countries namely Malaysia, Philippines, and Thailand. Southeast Asia is a region where its people share their similarities in term of geographical location, tradition, and culture in their ways of life. This study would definitely provide the new insight on toxoplasmosis in pregnant women.

## Materials and methods

**Figure 1:**
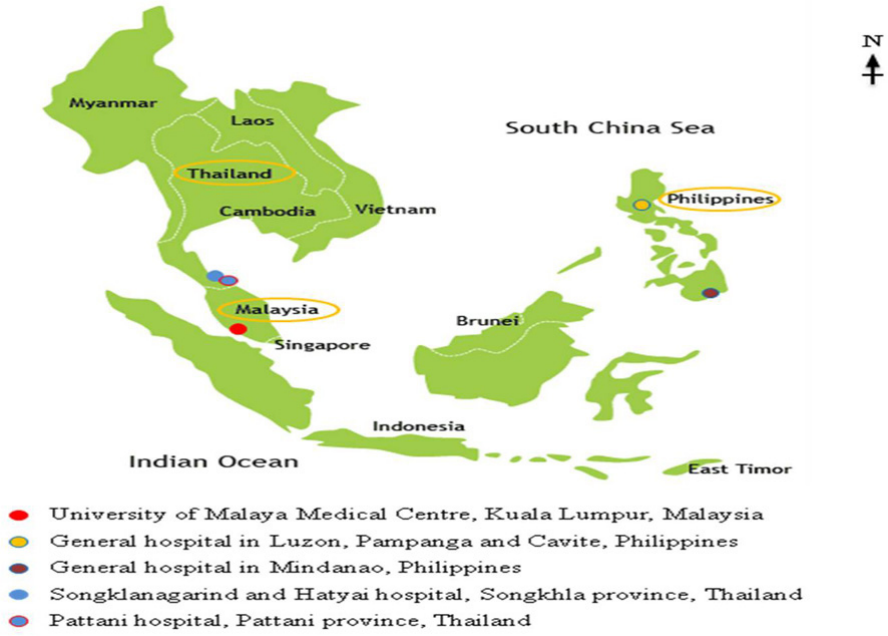
**Study sites in Malaysia, Philippines, and Thailand**.

This survey was carried out from January, 2012 to June, 2013 among 3 neighboring countries namely Malaysia, Philippines, and Thailand in Southeast Asia. A total of 2598 being 756 pregnant women visiting ANC at the Department of Obstetrics and Gynecology, University of Malaya Medical Centre, Kuala Lumpur, Malaysia, 1063 pregnant women visiting ANC at the general hospitals and in private clinics and lying-in/maternity hospitals in Luzon and Mindanao, the Philippines and 779 pregnant women visiting ANC at the Songklanagarind and Hatyai hospital, Songkhla province and Pattani hospital, Pattini province, Thailand were recruited (Figure 1). Inclusion criteria are pregnant women in any gestational periods, in any age groups and given verbal consent to participate in this study.

Demographic information and questions related to general knowledge about toxoplasmosis, risks factors, symptoms, and timing of infection, prevention knowledge, and preventive behavior was adopted from a previous study (Jones et al., 2003) with some modifications. Prior to answering questionnaires, these pregnant women were briefed about toxoplasmosis in term of the definition, risk factors, clinical features, preventive measures by medical practitioners and professional nurses. All data collected were entered and analyzed using SPSS version 17 (IL, Chicago, USA). Data with qualitative variables were expressed as frequency and percentage. Statistical analysis was performed using chi-square test as appropriate. A *P*-value of <0.05 was regarded as statistically significant.

## Results

A total of 2598 pregnant women participated in this survey with 756, 1063, and 779 from Malaysia, Philippines and Thailand, respectively. Majority of Malaysian pregnant women were in age group of 20–29 years, married, graduates, in their third trimester, expecting their first born, and had no history of abortion. Filipino pregnant women were mostly in age group of 20–29 years, married, were in secondary school leavers, in their second trimester, having = two children, and had no history of abortion. Meanwhile, Thai pregnant women were mostly in age group of 20–29 years, married, had tertiary level of education, in second trimester, have no children earlier, and had no history of abortion as shown in Table 1.

**Table 1:** **Demographic profiles of the pregnant women**.

**Factors**	**Sub-factor**	**Total (%)**	**Total (%)**		
			**Malaysia**	**Philippines**	**Thailand**
Age group	<20	123 (4.7)	5 (0.66)	57 (5.36)	61 (7.83)
	20–29	1323 (50.9)	**375 (49.60)**	**558 (52.49)**	**390 (50.06)**
	30–39	1025 (39.5)	353 (46.69)	385 (36.22)	287 (36.84)
	≥40	127 (4.9)	23 (3.04)	63 (5.93)	41 (5.26)
Marital status	Single	173 (6.7)	3 (0.40)	73 (6.87)	97 (12.45)
	Married	2425 (93.3)	**753 (99.60)**	**990 (93.13)**	**682 (87.55)**
Education	≤Primary	169 (6.5)	18 (2.38)	45 (4.23)	106 (13.61)
	Secondary	1342 (51.7)	268 (35.45)	**770 (72.44)**	304 (39.02)
	≥Tertiary	1087 (41.8)	**470 (62.17)**	248 (23.33)	**369 (47.37)**
Trimester	1st	690 (26.6)	151 (19.97)	368 (34.62	171 (21.95)
	2nd	990 (38.1)	235 (31.08)	**388 (36.50)**	**367 (47.11)**
	3rd	918 (35.3)	**370 (48.94)**	307 (28.88)	241 (30.94)
No. of children	None	952 (36.6)	**319 (42.20)**	294 (27.66)	**339 (43.52)**
	1	768 (29.6)	245 (32.41)	234 (22.01)	289 (37.10)
	≥2	878 (33.8)	192 (25.40)	**535 (50.33)**	151 (19.38)
History of abortion	Yes	250 (9.6)	148 (19.58)	8 (0.75)	94 (12.07)
	No	2348 (90.4)	**608 (80.42)**	**1055 (99.25)**	**685 (87.93)**
Total		2598	756	1063	779

The bold values indicate the highest number of pregnant women for each category from Malaysia, Philippines and Thailand, respectively.

In general, only 11% of these pregnant women had read, heard, or seen any information regarding toxoplasmosis and 3.5% of them were aware of being tested for the infection. A small percentage of these pregnant women knew that *Toxoplasma* were shed in the feces of infected cats (19.4%) and sometimes found in the raw or undercooked meat (11.0%). There was 16.1% of responding women knew that toxoplasmosis is caused by an infection, but 4.9% thought that it is caused by poison. Moreover, there was a low level of knowledge (<10%) about other risk factors such as consumption of undercooked meat, drinking untreated water and receiving blood transfusion. A high percentage of pregnant women indicated they do not eat undercooked meat and that they practice good hygienic measures such as washing their hands after handling raw meat, gardening, or changing cat litter as shown in Table 2.

**Table 2:**
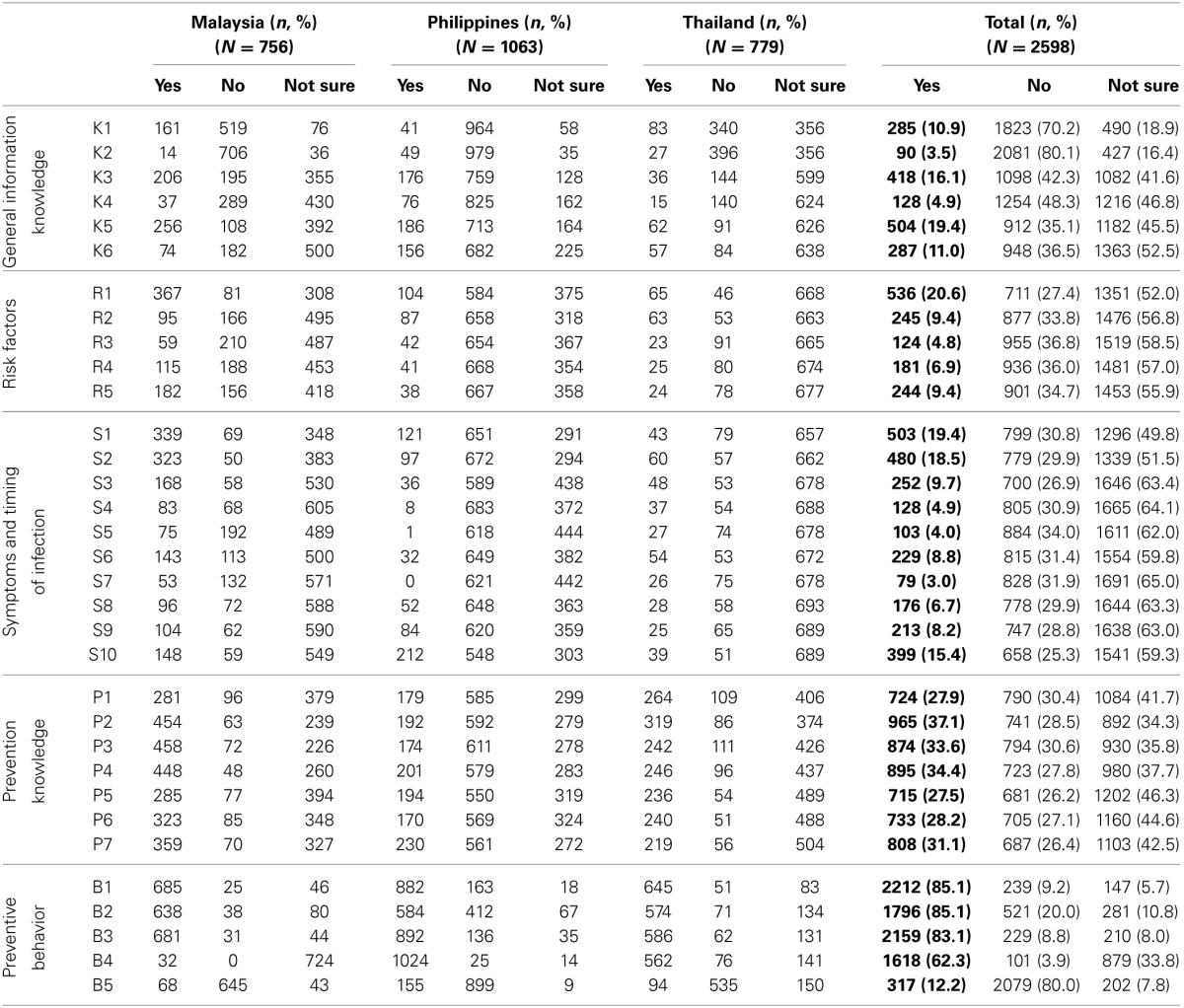
**Pregnant women responses for knowledge and practice on Toxoplasmosis by country**.

K1 to K6 are the knowledge on general information of toxoplasmosis. K1: Have you ever read, heard, or seen any information about toxoplasmosis. K2: Have you ever been tested for toxoplasmosis? K3: Is toxoplasmosis caused by an infection? K4: Is toxoplasmosis caused by a poison? K5: Is toxoplasmosis (T.gondii) shed in the feces of infected cats? K6: Is toxoplasmosis (T.gondii) sometimes found in raw or undercooked meat? R1 to R5 are the knowledge on risk factors of toxoplasmosis. R1: Can people get toxoplasmosis by changing cat litter? R2: Can people get toxoplasmosis by eating undercooked meat? R3: Can people get toxoplasmosis by receiving blood transfusion? R4: Can people get toxoplasmosis by drinking untreated water, e.g., rain, tap, or unboiled? R5: Can people get toxoplasmosis by gardening without gloves? S1 to S10 are the knowledge on symptoms and timing of infection of toxoplasmosis. S1: Can pregnant women develop serious complications after infection with toxoplasmosis (T.gondii)? S2: Can unborn and/or newborn children develop serious complications after infection with toxoplasmosis (T.gondii)? S3: Can toxoplasmosis in a pregnant women cause fever and feeling like you have the flu? S4: Can toxoplasmosis in a pregnant women cause swollen glands (lymph node)? S5: Can toxoplasmosis in a pregnant women cause no symptoms? S6: Toxoplasmosis (T.gondii) can only be passed from a pregnant woman to her fetus if she is newly infected during that pregnancy. S7: Toxoplasmosis (T.gondii) is rarely passed from a pregnant woman to her fetus if she is newly infected during that pregnancy. S8: A baby with toxoplasmosis may have no signs of illness at birth, but develop illness later. S9: A baby with toxoplasmosis may have vision problems. S10: A baby with toxoplasmosis may be treated with medicine. P1 to P7 are the preventive knowledge on ways to avoid toxoplasmosis. P1: Feeding your cat dry or commercial cat food and not letting it kill and eat rodents. P2: Avoiding stray cats. P3: Letting someone else change the cat's litter box. P4: Making sure the cat's litter box is changed daily. P5: Toxoplasmosis can be prevented by cooking meat well until no pink is seen and the juices run clear. P6: Toxoplasmosis can be prevented by thoroughly washing and/or peeling all fruits and vegetables before eating them. P7: Toxoplasmosis can be prevented by cleaning all cutting boards and utensils thoroughly after each use. B1 to B5 denotes the preventive behaviors of the pregnant women since becoming pregnant. B1: Do you routinely wash your hands after gardening? B2: Do you routinely wash your hands after changing cat litter? B3: Do you routinely wash your hands after handling raw meat? B4: Do you routinely wash your hands before eating food? B5: Do you eat raw meat?

The bold values indicate the total number of pregnant women who answered "Yes" for each questions in the questionnaire.

Table 3 shows the association between demographic profiles of pregnant women from each country with their preventive knowledge and preventive behavior on toxoplasmosis (Raw data can be found in Tables 3A,F from Supplement 1). Malaysian pregnant women in the age group of >40 years significantly avoiding stray cats; who had tertiary education significant association with their responses in feeding their cat with dry or commercial food, avoiding stray cats, let someone else changing cat litter box daily, wash cooking utensils after each use, washing hands after gardening, changing cat litter and handling raw meat while who educated from primary education significantly reported to wash their hand before eating and eat raw meat. Pregnant women who are in their first trimester significantly associated with avoiding stray cat and routinely wash hand before eating food while who are in their third trimester significantly practice in cooking meat well. Meanwhile, in Filipino pregnant women, only who graduate from primary level of education showed significant association with avoiding stray cats and who having ≥ two children significantly washing hands after handling raw meat. Thai pregnant women who in age group of 30–39 years indicated their significant association with prevention knowledge of washing fruits/vegetables before eating and clean cooking utensil after each use; age group <20 significantly associated with wash hand after changing cat litter, handling raw meat and before eating food; age group 20–29 years significantly eating raw meat. Married Thai pregnant women significantly associated with cooking meat well, washing fruits/vegetables before eating, cleaning cooking utensil after each use and washing hand before eating food while single pregnant women significantly eat raw meat. Thai pregnant women who were graduated from tertiary education or higher significantly associated with all questions ask when compare to other age group, including eating raw meat. Thai pregnant women who expecting their first child significantly associated with washing hand after changing cat litter, handling raw meat and before eating food. Only Thai pregnant women who had no history of abortion significantly associated with changed cat litter box daily.

**Table 3:**
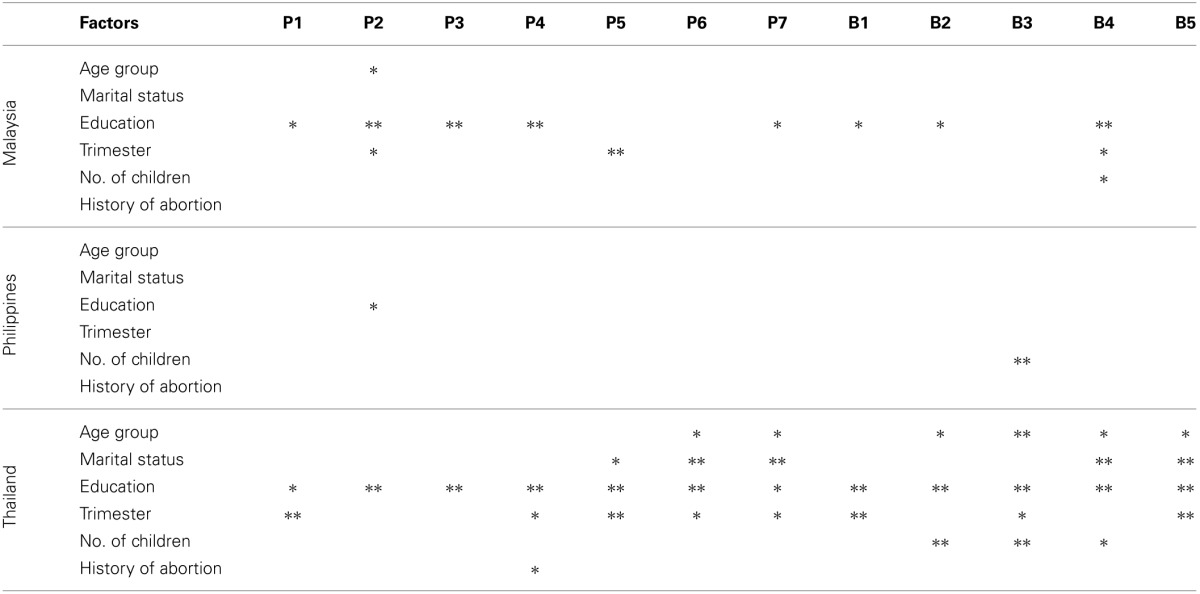
**Association between demographic profiles of pregnant women from each country with their preventive knowledge and preventive behavior on Toxoplasmosis**.

Significant association were tested by chi-square, ^*^p < 0.05; ^**^p < 0.01. P1 to P7 are the preventive knowledge on ways to avoid toxoplasmosis. P1: Feeding your cat dry or commercial cat food and not letting it kill and eat rodents. P2: Avoiding stray cats. P3: Letting someone else change the cat's litter box. P4: Making sure the cat's litter box is changed daily. P5: Toxoplasmosis can be prevented by cooking meat well until no pink is seen and the juices run clear. P6: Toxoplasmosis can be prevented by thoroughly washing and/or peeling all fruits and vegetables before eating them. P7: Toxoplasmosis can be prevented by cleaning all cutting boards and utensils thoroughly after each use. B1 to B5 denotes the preventive behaviors of the pregnant women since becoming pregnant. B1: Do you routinely wash your hands after gardening? B2: Do you routinely wash your hands after changing cat litter? B3: Do you routinely wash your hands after handling raw meat? B4: Do you routinely wash your hands before eating food? B5: Do you eat raw meat?

## Discussion

The prevalence of *Toxoplasma* infection varies among the three regions studied, namely 49% in Malaysia (Nissapatorn et al., 2003), 23.8% in the Philippines (Salibay et al., 2008), and 28.3% in Thailand (Nissapatorn et al., 2011). However, the knowledge about toxoplasmosis and its related preventive knowledge and behaviors may reduce the infection rate and its disease burden in pregnant women.

In the respondents' answers on the general knowledge of toxoplasmosis, majority of these pregnant women have no knowledge or unsure about this parasitic infection. This finding indicates the importance of educating the pregnant women with the preventive measures which have been highlighted in previous studies (Elsheikha, 2008; Costa et al., 2012; Amin et al., 2013). Malaysian and Thai pregnant women appear to have better precaution or preventive practice toward *Toxoplasma* infection when compared to their Filipino counterparts. This could be due to Malaysian and Thai pregnant women have higher education (≥tertiary level of education) which confirms their knowledge about toxoplasmosis.

Most of our subjects were not sure about the risk factors, symptoms, and timing of toxoplasmosis. Nevertheless, they routinely practice primary preventive behaviors, particularly in good hygienic condition against *Toxoplasma* infection. This evidence is supported by most of our pregnant women (>83%) wash their hands after gardening, changing cat litter and handling raw meat. *Toxoplasma gondii* poses a public health problem for both infection rate and disease burden that have been reported in pregnant women from different parts of the world. This parasite has been found in any given environment conditions that contaminating with cat's feces. Approximately 1.2 million metric tons of cat feces were being deposited in the environment annually with the oocysts burden measured in a community survey was 3 to 434 oocysts per square foot in USA (Torrey and Yolken, 2013) and there have been reports showing that having a close contact with cats and cleaning their litter may transmit the disease to pregnant women (Fakhfakh et al., 2013). Exposure to infected cats by these women could lead to severe outcomes to her carried fetus. Many studies reported *Toxoplasma* infection in animals for human consumption (Bangoura et al., 2013; Hill and Dubey, 2013; Kang et al., 2013; Lopes et al., 2013), 12.2% of pregnant women who consume raw meat especially Malaysian who were graduate from primary school and Thai's who were in age group 20–29, single, graduate from tertiary education and in their first semester of pregnancy could be at risk for *Toxoplasma* infection.

Further analysis revealed that there were some significant association found between the demographic profiles and the responses from these pregnant women regarding their prevention knowledge and preventive behavior. Many of these pregnant women (>30%) know that by avoiding stray cats, allowing someone else to change the cat's litter box, making sure the cat's litter box is changed daily, cleaning cooking utensil after each use are preventive measures of toxoplasmosis. Most of them (>80%) routinely wash their hand after gardening, changing cat litter and after handling raw meat indicated their constantly practice basic personal hygiene during their pregnancy. Looking at pregnant women in the Philippines, it was found no significant association between demographic profiles and their knowledge on toxoplasmosis, except for their level of education and the number of children. Overall, level of education, marital status, trimester of pregnancy and the number of children play an important role in preventing *Toxoplasma* infection found in these pregnant women. History of abortion did not have any significant association except in Thai pregnant women who were changing cat's litter box daily.

This study highlights the level of knowledge and practice on awareness of *Toxoplasma* infection among the pregnant women from three Southeast Asian countries: Malaysia, Philippines, and Thailand. The findings of this study provide vital information in better understanding about the knowledge and practice of toxoplasmosis among pregnant women in this region. Therefore, it emphasizes the need for implementation of health education among this target group to further educate them on the preventive and control measures. We strongly believe that with adequate knowledge and awareness through health education on toxoplasmosis will remarkably eliminate the infection rate and subsequently eradicate its disease burden in these countries. The obtained data also coincide with the actual situation that the incidence of primary acute toxoplasmosis is very low and also clinical evidence of congenital toxoplasmosis is rarely reported in this region. Our finding is supported by a previous study in Belgium showed that there was a significant decrease in the incidence of *Toxoplasma* seroconversion after the introduction of intensive counseling for pregnant women about toxoplasmosis (Gollub et al., 2008). Other previous studies also highlight the importance of health education among the pregnant women in order to reduce the seroprevalence of this disease hence minimizing the adverse effects of infection in the fetus or newborn (Fonseca et al., 2012; Amin et al., 2013; Pereboom et al., 2013). Unfortunately, it was found that some physicians, obstetrics, and medical staffs had lack of knowledge on this parasitic infection thus failed to provide sufficient information the pregnant women (Ziemba et al., 2010; Alvarado-Esquivel et al., 2011; da Silva et al., 2011). Therefore, medical personnel should be educated on the level of knowledge and practice of toxoplasmosis, subsequently, an appropriate health education could then be provided to pregnant women and the healthcare related staffs to better understand manifestation of this parasitic infection.

## Conclusions

In this study, the knowledge and practices on toxoplasmosis among the pregnant women from Malaysia, Philippines, and Thailand were studied. Of the respondents, a substantial part did have knowledge about preventive practices to avoid toxoplasmosis during their pregnancy. Advising pregnant women about their healthy lifestyle and practices of good preventive measures to prevent this parasitic disease remain crucial. Awareness and education about this parasitic infection in pregnant woman would be helpful in preventing disease transmission and the incidence of clinical outcomes in their carried fetus or newborns. In accordance with this study, we have implemented healthcare education programme related to *Toxoplasma* infection in pregnant women by distribution of brochures in the study areas; Malaysia, Philippines and Thailand and in their respective national languages (Brochures included as Supplement 2).

## Author contributions

Cristina C. Salibay, Nongyao Sawangjaroen, and Veeranoot Nissapatorn designed the study. Cristina C. Salibay, Julieta Z. Dungca, Mary Mae M. Cheung, Nongyao Sawangjaroen, and Si-Lay Khaing carried out the experiment, Hemah Andiappan contributed most on manuscript writing. Cristina C. Salibay, Hemah Andiappan, Nongyao Sawangjaroen, Veeranoot Nissapatorn, Waenurama Chemoh, and Ching Xiao Teng helped in manuscript writing and editing. Cristina C. Salibay, Julieta Z. Dungca, Mary Mae M. Cheung, Noor A. Mat Adenan, Nongyao Sawangjaroen, Si-Lay Khaing, Yee-Ling Lau and Veeranoot Nissapatorn provided opinions and suggestions about this manuscript. All authors read and approved the final version of the manuscript.

### Conflict of interest statement

The authors declare that the research was conducted in the absence of any commercial or financial relationships that could be construed as a potential conflict of interest.
