# Supplementary material for: Knowledge and practice on Toxoplasma infection in pregnant women from Malaysia, Philippines, and Thailand
Source: Front Microbiol. 2014 Jun 11;5:291. doi: 10.3389/fmicb.2014.00291 (PMC4052801; doi:10.3389/fmicb.2014.00291)
Supplement: Supplementary file 1 [file DataSheet1.ZIP › Supp Table 1.DOC]

**Supplement 1**

**Table 3a:- Association** between demographic profiles of Malaysian pregnant women with their preventive knowledge of toxoplasmosis.

| Factors | | Total (%) | Total (%) | | | | | | |
| --- | --- | --- | --- | --- | --- | --- | --- | --- | --- |
|  |  |  | P1 | P2 | P3 | P4 | P5 | P6 | P7 |
| Age Group | <20 | 5 (0.7) | 1 (20.0) | 2 (40.0) | 2 (40.0) | 3 (60.0) | 2 (40.0) | 2 (40.0) | 1 (20.0) |
|  | 20-29 | 375 (49.6) | 142 (37.9) | 232 (61.9) | 236 (62.9) | 218 (58.1) | 140 (37.3) | 164 (43.7) | 175 (46.7) |
|  | 30-39 | 353 (46.7) | 125 (35.4) | 203 (57.5) | 204 (57.8) | 209 (59.2) | 130 (36.8) | 143 (40.5) | 169 (47.9) |
|  | ≥40 | 23(3.0) | 13 (56.5) | **17 (73.9)** | 16 (69.6) | 18 (78.3) | 13 (56.5) | 14 (60.9) | 14 (60.9) |
|  |  | *P*-value | 0.524 | **0.043** | 0.687 | 0.106 | 0.432 | 0.425 | 0.625 |
| Marital status | Single | 3 (0.4) | 0 | 1 (33.3) | 1 (33.3) | 2 (66.7) | 2 (66.7) | 0 | 1 (33.3) |
|  | Married | 753 (99.6) | 281 (37.3) | 453 (60.2) | 457 (60.7) | 446 (59.2) | 283 (37.6) | 323 (42.9) | 358 (47.5) |
|  |  | *P*-value | 0.125 | 0.404 | 0.364 | 0.815 | 0.499 | 0.097 | 0.599 |
| Education | ≤Primary | 18 (2.4) | 2 (11.1) | 6 (33.3) | 6 (33.3) | 7 (38.9) | 4 (22.2) | 6 (33.3) | 8 (44.4) |
|  | Secondary | 268 (35.5) | 93 (34.7) | 142 (53.0) | 149 (55.6) | 146 (54.5) | 91 (34.0) | 111 (41.4) | 114 (42.5) |
|  | ≥Tertiary | 470 (62.2) | **186 (39.6)** | **306 (65.1)** | **303 (64.5)** | **295 (62.8)** | 190 (40.4) | 206 (43.8) | **237 (50.4)** |
|  |  | *P*-value | **0.012** | **0.001** | **0.007** | **0.006** | 0.058 | 0.401 | **0.033** |
| Trimester | 1st | 151 (20.0) | 64 (42.4) | **94 (62.3)** | 98 (64.9) | 100 (66.2) | 56 (37.1) | 66 (43.7) | 70 (46.4) |
|  | 2nd | 235 (31.1) | 89 (37.9) | 143 (60.9) | 142 (60.4) | 136 (57.9) | 86 (36.6) | 91 (38.7) | 108 (46.0) |
|  | 3rd | 370 (48.9) | 128 (34.6) | 217 (58.6) | 218 (58.9) | 212 (57.3) | **143 (38.6)** | 166 (44.9) | 181 (48.9) |
|  |  | *P*-value | 0.477 | **0.019** | 0.283 | 0.147 | **0.004** | 0.289 | 0.063 |
| No. of Children | None | 319 (42.2) | 122 (38.2) | 189 (59.2) | 200 (62.7) | 195 (61.1) | 116 (36.4) | 136 (42.6) | 150 (47.0) |
|  | 1 | 245 (32.4) | 91 (37.1) | 157 (64.1) | 151 (61.6) | 147 (60.0) | 96 (39.2) | 103 (42.0) | 120 (49.0) |
|  | ≥2 | 192 (25.4) | 68 (35.4) | 108 (56.3) | 107 (55.7) | 106 (55.2) | 73 (38.0) | 84 (43.8) | 89 (46.4) |
|  |  | *P*-value | 0.140 | 0.390 | 0.528 | 0.703 | 0.742 | 0.948 | 0.793 |
| History of abortion | Yes | 148 (19.6) | 53 (35.8) | 81 (54.7) | 80 (54.1) | 85 (57.4) | 57 (38.5) | 63 (42.6) | 70 (47.3) |
|  | No | 608 (80.42) | 228 (37.5) | 373 (61.0) | 378 (62.2) | 363 (59.7) | 228 (37.5) | 260 (42.8) | 289 (47.5) |
|  |  | *P*-value | 0.873 | 0.427 | 0.394 | 0.932 | 0.486 | 0.536 | 0.768 |

Note: **P1** to **P7** are the preventive knowledge on ways to avoid toxoplasmosis. **P1**: Feeding your cat dry or commercial cat food and not letting it kill and eat rodents. **P2**: Avoiding stray cats. **P3**: Letting someone else change the cat's litter box. **P4**: Making sure the cat's litter box is changed daily. **P5**: Toxoplasmosis can be prevented by cooking meat well until no pink is seen and the juices run clear. **P6**: Toxoplasmosis can be prevented by thoroughly washing and/or peeling all fruits and vegetables before eating them. **P7**: Toxoplasmosis can be prevented by cleaning all cutting boards and utensils thoroughly after each use.

The “**BOLD**” figures in the table were the category of the factors which shown significant association (*P*-value <0.05) with the preventive knowledge.

**Table 3b:- Association between demographic profiles of Philippines** pregnant women with their preventive knowledge of toxoplasmosis.

| Factors | | Total (%) | Total (%) | | | | | | |
| --- | --- | --- | --- | --- | --- | --- | --- | --- | --- |
|  |  |  | P1 | P2 | P3 | P4 | P5 | P6 | P7 |
| Age Group | <20 | 57 (5.4) | 9 (15.8) | 11 (19.3) | 10 (17.5) | 10 (17.5) | 10 (17.5) | 8 (14.0) | 13 (22.8) |
|  | 20-29 | 558 (52.5) | 96 (17.2) | 101 (18.1) | 88 (15.8) | 108 (19.4) | 106 (19.0) | 92 (16.5) | 126 (22.6) |
|  | 30-39 | 385 (36.2) | 65 (16.9) | 69 (17.9) | 66 (17.1) | 71 (18.4) | 67 (17.4) | 60 (15.6) | 79 (20.5) |
|  | ≥40 | 63 (5.9) | 9 (14.3) | 11 (17.5) | 10 (15.9) | 12 (19.0) | 11 (17.5) | 10 (15.9) | 12 (19.0) |
|  |  | *P*-value | 0.336 | 0.143 | 0.469 | 0.310 | 0.176 | 0.190 | 0.119 |
| Marital status | Single | 73 (6.9) | 18 (24.7) | 20 (27.4) | 19 (26.0) | 21 (28.8) | 18 (24.7) | 16 (21.9) | 22 (30.1) |
|  | Married | 990 (93.1) | 161 (16.3) | 172 (17.4) | 155 (15.7) | 180 (18.2) | 176 (17.8) | 154 (15.6) | 208 (21.0) |
|  |  | *P*-value | 0.163 | 0.085 | 0.067 | 0.070 | 0.340 | 0.358 | 0.185 |
| Education | ≤Primary | 45 (4.2) | 12 (26.7) | **12 (26.7)** | 9 (20.0) | 10 (22.2) | 10 (22.2) | 9 (20.0) | 11 (24.4) |
|  | Secondary | 770 (72.4) | 122 (15.8) | 127 (16.5) | 117 (15.2) | 135 (17.5) | 131 (17.0) | 112 (14.5) | 157 (20.4) |
|  | ≥Tertiary | 248 (23.3) | 45 (18.2) | 53 (21.4) | 48 (19.4) | 56 (22.6) | 53 (21.4) | 49 (19.8) | 62 (25.0) |
|  |  | *P*-value | 0.183 | **0.049** | 0.178 | 0.108 | 0.065 | 0.085 | 0.295 |
| Trimester | 1st | 368 (34.6) | 60 (16.3) | 67 (18.2) | 61 (16.6) | 69 (18.8) | 70 (19.0) | 67 (18.2) | 80 (21.7) |
|  | 2nd | 388 (36.5) | 75 (19.3) | 77 (19.8) | 70 (18.0) | 81 (20.9) | 77 (19.8) | 65 (16.8) | 91 (23.5) |
|  | 3rd | 307 (28.9) | 44 (14.3) | 48 (15.6) | 43 (14.0) | 51 (16.6) | 47 (15.3) | 38 (12.4) | 59 (19.2) |
|  |  | *P*-value | 0.147 | 0.446 | 0.405 | 0.462 | 0.317 | 0.066 | 0.556 |
| No. of Children | None | 294 (27.7) | 51 (17.3) | 55 (18.7) | 50 (17.0) | 57 (19.4) | 55 (18.7) | 49 (16.7) | 66 (22.4) |
|  | 1 | 234 (22.0) | 28 (12.0) | 30 (12.8) | 29 (12.4) | 33 (14.1) | 32 (13.7) | 30 (12.8) | 39 (16.7) |
|  | ≥2 | 535 (50.3) | 100 (18.7) | 107 (20.0) | 95 (17.8) | 111 (20.7) | 107 (20.0) | 91 (17.0) | 125 (23.4) |
|  |  | *P*-value | 0.159 | 0.176 | 0.218 | 0.179 | 0.198 | 0.410 | 0.206 |
| History of abortion | Yes | 8 (0.8) | 0 | 1 (12.5) | 1 (12.5) | 1 (12.5) | 0 | 1 (12.5) | 0 |
|  | No | 1055 (99.3) | 179 (17.0) | 191 (18.1) | 173 (16.4) | 200 (19.0) | 194 (18.4) | 169 (16.0) | 230 (21.8) |
|  |  | *P*-value | 0.145 | 0.761 | 0.774 | 0.761 | 0.147 | 0.902 | 0.084 |

Note: **P1** to **P7** are the preventive knowledge on ways to avoid toxoplasmosis. **P1**: Feeding your cat dry or commercial cat food and not letting it kill and eat rodents. **P2**: Avoiding stray cats. **P3**: Letting someone else change the cat's litter box. **P4**: Making sure the cat's litter box is changed daily. **P5**: Toxoplasmosis can be prevented by cooking meat well until no pink is seen and the juices run clear. **P6**: Toxoplasmosis can be prevented by thoroughly washing and/or peeling all fruits and vegetables before eating them. **P7**: Toxoplasmosis can be prevented by cleaning all cutting boards and utensils thoroughly after each use.

The “**BOLD**” figures in the table were the category of the factors which shown significant association (*P*-value <0.05) with the preventive knowledge.

**Table 3c:- Association between demographic profiles of Thailand pregnant women with their preventive knowledge of toxoplasmosis.**

| Factors | | Total (%) | Total (%) | | | | | | |
| --- | --- | --- | --- | --- | --- | --- | --- | --- | --- |
|  |  |  | P1 | P2 | P3 | P4 | P5 | P6 | P7 |
| Age Group | <20 | 61 (7.83) | 13 (21.3) | 17 (27.9) | 15 (24.6) | 13 (21.3) | 12 (19.7) | 8 (13.1) | 6 (9.8) |
|  | 20-29 | 390 (50.06) | 135 (34.6) | 154 (39.5) | 132 (33.8) | 127 (32.6) | 118 (30.3) | 118 (30.3) | 108 (27.7) |
|  | 30-39 | 287 (36.84) | 99 (34.5) | 132 (46.0) | 85 (29.6) | 95 (33.10) | 94 (32.8) | **104 (36.2)** | **94 (32.8)** |
|  | ≥40 | 41 (5.26) | 17 (41.5) | 16 (39.0) | 10 (24.4) | 11 (26.8) | 12 (29.3) | 10 (24.4) | 11 (26.8) |
|  |  | *P*-value | 0.389 | 0.249 | 0.610 | 0.499 | 0.457 | **0.018** | **0.031** |
| Marital status | Single | 97 (12.45) | 33 (34.0) | 35 (36.1) | 28 (28.9) | 30 (30.9) | 29 (29.9) | 28 (28.9) | 26 (26.8) |
|  | Married | 682 (87.55) | 231 (33.9) | 284 (41.6) | 214 (31.4) | 216 (31.7) | **207 (30.4)** | **212 (31.1)** | **193 (28.3)** |
|  |  | *P*-value | 0.893 | 0.397 | 0.426 | 0.796 | **0.025** | **0.001** | **0.003** |
| Education | ≤Primary | 106 (13.61) | 24 (22.6) | 23 (21.7) | 19 (17.9) | 17 (16.0) | 20 (18.87) | 21 (19.8) | 19 (17.9) |
|  | Secondary | 304 (39.02) | 98 (32.2) | 124 (40.8) | 87 (28.6) | 82 (27.0) | 81 (26.6) | 83 (27.3) | 79 (26.0) |
|  | ≥Tertiary | 369 (47.37) | **142 (38.5)** | **172 (46.6)** | **136 (36.9)** | **147 (39.8)** | **135 (36.6)** | **136 (36.9)** | **121 (32.8)** |
|  |  | *P*-value | **0.041** | **0.000** | **0.001** | **0.000** | **0.000** | **0.001** | **0.015** |
| Trimester | 1st | 171 (21.95) | **73 (42.7)** | 74 (43.3) | 63 (36.8) | **60 (35.1)** | **70 (40.9)** | **65 (38.0)** | **61 (35.7)** |
|  | 2nd | 367 (47.11) | 123 (33.5) | 144 (39.2) | 102 (27.8) | 113 (30.8) | 110 (30.0) | 118 (32.2) | 101 (27.5) |
|  | 3rd | 241 (30.94) | 68 (28.2) | 101 (41.9) | 77 (32.0) | 73 (30.3) | 56 (23.2) | 57 (23.7) | 57 (23.7) |
|  |  | *P*-value | **0.000** | 0.177 | 0.147 | **0.011** | **0.003** | **0.022** | **0.037** |
| No. of Children | None | 339 (43.52) | 122 (36.0) | 132 (38.9) | 112 (33.0) | 111 (32.7) | 113 (33.3) | 104 (30.7) | 93 (27.4) |
|  | 1 | 289 (37.10) | 97 (33.6) | 132 (45.7) | 89 (30.8) | 90 (31.1) | 85 (29.4) | 97 (33.6) | 87 (30.1) |
|  | ≥2 | 151 (19.38) | 45 (29.8) | 55 (36.4) | 41 (27.2) | 45 (29.8) | 38 (25.2) | 39 (25.8) | 39 (25.8) |
|  |  | *P*-value | 0.513 | 0.210 | 0.781 | 0.668 | 0.383 | 0.379 | 0.789 |
| History of abortion | Yes | 94 (12.07) | 25 (26.6) | 40 (42.6) | 26 (27.7) | 25 (26.6) | 21 (22.3) | 24 (25.5) | 23 (24.5) |
|  | No | 685 (87.93) | 239 (34.9) | 279 (40.7) | 216 (31.5) | **221 (32.3)** | 215 (31.4) | 216 (31.5) | 196 (28.6) |
|  |  | *P*-value | 0.134 | 0.505 | 0.821 | **0.031** | 0.109 | 0.270 | 0.583 |

Note: **P1** to **P7** are the preventive knowledge on ways to avoid toxoplasmosis. **P1**: Feeding your cat dry or commercial cat food and not letting it kill and eat rodents. **P2**: Avoiding stray cats. **P3**: Letting someone else change the cat's litter box. **P4**: Making sure the cat's litter box is changed daily. **P5**: Toxoplasmosis can be prevented by cooking meat well until no pink is seen and the juices run clear. **P6**: Toxoplasmosis can be prevented by thoroughly washing and/or peeling all fruits and vegetables before eating them. **P7**: Toxoplasmosis can be prevented by cleaning all cutting boards and utensils thoroughly after each use.

The “**BOLD**” figures in the table were the category of the factors which shown significant association (*P*-value <0.05) with the preventive knowledge.

**Table 3d:- Association** between demographic profiles of Malaysian pregnant women with their practice in preventive behavior of toxoplasmosis since becoming pregnant.

| Factors | | Total (%) | Total (%) | | | | |
| --- | --- | --- | --- | --- | --- | --- | --- |
|  |  |  | B1 | B2 | B3 | B4 | B5 |
| Age Group | <20 | 5 (0.7) | 5 (100.0) | 4 (80.0) | 5 (100.0) | 1 (20.0) | 0 |
|  | 20-29 | 375 (49.6) | 340 (90.7) | 318 (84.8) | 334 (89.1) | 11 (2.9) | 32 (8.5) |
|  | 30-39 | 353 (46.7) | 319 (90.4) | 298 (84.4) | 320 (90.7) | 20 (5.7) | 36 (10.2) |
|  | ≥40 | 23 (3.0) | 21 (91.3) | 18 (78.3) | 22 (95.7) | 0 | 0 |
|  |  | *P*-value | 0.774 | 0.394 | 0.185 | 0.071 | 0.116 |
| Marital status | Single | 3 (0.4) | 2 (66.7) | 2 (66.7) | 2 (66.7) | 0 | 0 |
|  | Married | 753 (99.6) | 683 (90.7) | 636 (84.5) | 679 (90.2) | 32 (4.2) | 68 (9.0) |
|  |  | *P*-value | 0.335 | 0.507 | 0.316 | 1.000 | 0.277 |
| Education | ≤Primary | 18 (2.4) | 14 (77.8) | 13 (72.2) | 16 (88.9) | **6 (33.3)** | **2 (11.1)** |
|  | Secondary | 268 (35.5) | 233 (86.9) | 214 (79.9) | 233 (86.9) | 10 (3.7) | 22 (8.2) |
|  | ≥Tertiary | 470 (62.2) | **438 (93.2)** | **411 (87.4)** | **432 (91.9)** | 16 (3.4) | 44 (9.4) |
|  |  | *P*-value | **0.029** | **0.021** | 0.226 | **0.000** | 0.568 |
| Trimester | 1st | 151 (20.0) | 133 (88.1) | 124 (82.1) | 132 (87.4) | **10 (6.6)** | 12 (7.9) |
|  | 2nd | 235 (31.1) | 215 (91.5) | 196 (83.4) | 211 (89.8) | 13 (5.5) | 22 (9.4) |
|  | 3rd | 370 (48.9) | 337 (91.1) | 318 (85.9) | 338 (91.4) | 9 (2.4) | 34 (9.2) |
|  |  | *P*-value | 0.534 | 0.080 | 0.081 | **0.048** | 0.951 |
| No. of Children | None | 319 (42.2) | 296 (92.8) | 278 (87.1) | 288 (90.3) | 17 (5.3) | 27 (8.5) |
|  | 1 | 245 (32.4) | 218 (89.0) | 199 (81.2) | 218 (89.0) | **14 (5.7)** | 27(11.0) |
|  | ≥2 | 192 (25.4) | 171 (89.1) | 161 (83.9) | 175 (91.1) | 1 (0.5) | 14 (7.3) |
|  |  | *P*-value | 0.292 | 0.412 | 0.716 | **0.012** | 0.648 |
| History of abortion | Yes | 148 (19.6) | 135 (91.2) | 121 (81.8) | 133 (89.9) | 3 (2.0) | 15 (10.1) |
|  | No | 608 (80.4) | 550 (90.5) | 517 (85.0) | 548 (90.1) | 29 (4.8) | 53 (8.7) |
|  |  | *P*-value | 0.403 | 0.820 | 0.995 | 0.263 | 0.961 |

Note: **B1** to **B5** denotes the preventive behaviors of the pregnant women since becoming pregnant. **B1**: Do you routinely wash your hands after gardening? **B2**: Do you routinely wash your hands after changing cat litter? **B3**: Do you routinely wash your hands after handling raw meat? **B4**: Do you routinely wash your hands before eating food? **B5**: Do you eat raw meat?

The “**BOLD**” figures in the table were the category of the factors which shown significant association (*P*-value <0.05) with the preventive behaviors.

**Table 3e:- Association between demographic profiles of Philippines** pregnant women with their practice in preventive behavior of toxoplasmosis since becoming pregnant.

| Factors | | Total (%) | Total (%) | | | | |
| --- | --- | --- | --- | --- | --- | --- | --- |
|  |  |  | B1 | B2 | B3 | B4 | B5 |
| Age Group | <20 | 57 (5.4) | 52 (91.2) | 37 (64.9) | 49 (86.0) | 54 (94.7) | 10 (17.5) |
|  | 20-29 | 558 (52.5) | 456 (81.7) | 290 (52.0) | 469 (84.1) | 537 (96.2) | 72 (12.9) |
|  | 30-39 | 385 (36.2) | 323 (83.9) | 222 (57.7) | 323 (83.9) | 373 (96.9) | 60 (15.6) |
|  | ≥40 | 63 (5.9) | 51 (81.0) | 35 (55.6) | 51 (81.0) | 60 (95.2) | 13 (20.6) |
|  |  | *P*-value | 0.546 | 0.409 | 0.928 | 0.563 | 0.250 |
| Marital status | Single | 73 (6.9) | 60 (82.2) | 41 (56.2) | 62 (84.9) | 69 (94.5) | 11 (15.1) |
|  | Married | 990 (93.1) | 822 (83.0) | 543 (54.8) | 830 (83.8) | 955 (96.5) | 144 (14.5) |
|  |  | *P*-value | 0.773 | 0.713 | 0.411 | 0.603 | 0.184 |
| Education | ≤Primary | 45 (4.2) | 37 (82.2) | 24 (53.3) | 38 (84.4) | 39 (86.7) | 8 (17.8) |
|  | Secondary | 770 (72.4) | 633 (82.2) | 415 (53.9) | 633 (82.2) | 746 (96.9) | 117 (15.2) |
|  | ≥Tertiary | 248 (23.3) | 212 (85.5) | 145 (58.5) | 221 (89.1) | 239 (96.4) | 30 (12.1) |
|  |  | *P*-value | 0.690 | 0.224 | 0.054 | 0.183 | 0.129 |
| Trimester | 1st | 368 (34.6) | 303 (82.3) | 191 (51.9) | 302 (82.1) | 353 (95.9) | 45 (12.2) |
|  | 2nd | 388 (36.5) | 327 (84.3) | 228 (58.8) | 327 (84.3) | 371 (95.6) | 63 (16.2) |
|  | 3rd | 307 (28.9) | 252 (82.1) | 165 (53.7) | 263 (85.7) | 300 (97.7) | 47 (15.3) |
|  |  | *P*-value | 0.114 | 0.245 | 0.726 | 0.590 | 0.366 |
| No. of Children | None | 294 (27.7) | 249 (84.7) | 163 (55.4) | 230 (78.2) | 281 (95.6) | 33 (11.2) |
|  | 1 | 234 (22.0) | 191 (81.6) | 130 (55.6) | 189 (80.8) | 224 (95.7) | 41 (17.5) |
|  | ≥2 | 535 (50.3) | 442 (82.6) | 291 (54.4) | **473 (88.4)** | 519 (97.0) | 81 (15.1) |
|  |  | *P*-value | 0.764 | 0.675 | **0.001** | 0.097 | 0.180 |
| History of abortion | Yes | 8 (0.8) | 8 (100.0) | 4 (50.0) | 6 (75.0) | 8 (100.0) | 1 (12.5) |
|  | No | 1055 (99.3) | 874 (82.8) | 580 (55.0) | 886 (84.0) | 1016 (96.3) | 154 (14.6) |
|  |  | *P*-value | 0.223 | 0.525 | 0.511 | 0.741 | 0.154 |

Note: **B1** to **B5** denotes the preventive behaviors of the pregnant women since becoming pregnant. **B1**: Do you routinely wash your hands after gardening? **B2**: Do you routinely wash your hands after changing cat litter? **B3**: Do you routinely wash your hands after handling raw meat? **B4**: Do you routinely wash your hands before eating food? **B5**: Do you eat raw meat?

The “**BOLD**” figures in the table were the category of the factors which shown significant association (*P*-value <0.05) with the preventive behaviors.

**Table 3f:- Association between demographic profiles of Thailand** pregnant women with their practice in preventive behavior of toxoplasmosis since becoming pregnant.

| Factors | | Total (%) | Total (%) | | | | |
| --- | --- | --- | --- | --- | --- | --- | --- |
|  |  |  | B1 | B2 | B3 | B4 | B5 |
| Age Group | <20 | 61 (7.83) | 52 (85.2) | **51 (83.6)** | **54 (88.5)** | **49 (80.3)** | 8 (13.1) |
|  | 20-29 | 390 (50.06) | 324 (83.1) | 296 (75.9) | 302 (77.4) | 285 (73.1) | **57 (14.6)** |
|  | 30-39 | 287 (36.84) | 236 (82.2) | 201 (70.0) | 205 (71.4) | 202 (70.4) | 26 (9.1) |
|  | ≥40 | 41 (5.26) | 33 (80.5) | 26 (63.4) | 25 (61.0) | 26 (63.4) | 3 (7.3) |
|  |  | *P*-value | 0.057 | **0.020** | **0.001** | **0.026** | **0.023** |
| Marital status | Single | 97 (12.45) | 79 (81.4) | 77 (79.4) | 77 (79.4) | 69 (71.1) | **24 (24.7)** |
|  | Married | 682 (87.55) | 566 (83.0) | 497 (72.9) | 509 (74.6) | **493 (72.3)** | 70 (10.3) |
|  |  | *P*-value | 0.476 | 0.077 | 0.061 | **0.002** | **0.000** |
| Education | ≤Primary | 106 (13.61) | 84 (79.2) | 67 (63.2) | 73 (68.9) | 70 (6.6) | 4 (3.8) |
|  | Secondary | 304 (39.02) | 247 (81.3) | 218 (71.7) | 229 (75.3) | 210 (69.1) | 31 (10.2) |
|  | ≥Tertiary | 369 (47.37) | **314 (85.1)** | **289 (78.3)** | **284 (77.0)** | **282 (76.4)** | **59 (16.0)** |
|  |  | *P*-value | **0.000** | **0.000** | **0.000** | **0.001** | **0.002** |
| Trimester | 1st | 171 (21.95) | 131 (76.6) | 121 (70.8) | 114 (66.7) | 110 (64.3) | **33 (19.3)** |
|  | 2nd | 367 (47.11) | 297 (80.9) | 277 (75.5) | 280 (76.3) | 268 (73.0) | 35 (9.5) |
|  | 3rd | 241 (30.94) | **217 (90.0)** | 176 (73.0) | **192 (79.7)** | 184 (76.3) | 26 (10.8) |
|  |  | *P*-value | **0.006** | 0.538 | **0.049** | 0.093 | **0.000** |
| No. of Children | None | 339 (43.52) | 287 (84.7) | **270 (79.6)** | **274 (80.8)** | **262 (77.3)** | 39 (11.5) |
|  | 1 | 289 (37.10) | 232 (80.3) | 193 (66.8) | 198 (68.5) | 191 (66.1) | 41 (14.2) |
|  | ≥2 | 151 (19.38) | 126 (83.4) | 111 (73.5) | 114 (75.5) | 109 (72.2) | 14 (9.3) |
|  |  | *P*-value | 0.467 | **0.005** | **0.004** | **0.021** | 0.204 |
| History of abortion | Yes | 94 (12.07) | 81 (86.2) | 70 (74.5) | 71 (75.5) | 66 (70.2) | 9 (9.6) |
|  | No | 685 (87.93) | 564 (82.3) | 504 (73.6) | 515 (75.2) | 496 (72.4) | 85 (12.4) |
|  |  | *P*-value | 0.398 | 0.283 | 0.885 | 0.989 | 0.615 |

Note: **B1** to **B5** denotes the preventive behaviors of the pregnant women since becoming pregnant. **B1**: Do you routinely wash your hands after gardening? **B2**: Do you routinely wash your hands after changing cat litter? **B3**: Do you routinely wash your hands after handling raw meat? **B4**: Do you routinely wash your hands before eating food? **B5**: Do you eat raw meat?

The “**BOLD**” figures in the table were the category of the factors which shown significant association (*P*-value <0.05) with the preventive behaviors
