# Supplementary material for: Knowledge and practice on Toxoplasma infection in pregnant women from Malaysia, Philippines, and Thailand
Source: Front Microbiol. 2014 Jun 11;5:291. doi: 10.3389/fmicb.2014.00291 (PMC4052801; doi:10.3389/fmicb.2014.00291)
Supplement: Supplementary file 1 [file DataSheet1.ZIP › Supp Table 2.DOC]

Supplement 2: Health care education on Toxoplasmosis in pregnant women.

Brochures in English Language (General)

Brochures in Malay Language (Malaysia)

Brochures in Filipino language (Philippines)

Brochures in Thai language (Thailand)
